# Nationwide decline in psychiatric hospitalizations and costs in Brazil, 2008–2022: a retrospective descriptive study evidencing reform-driven community mental health gains

**DOI:** 10.1016/j.lana.2026.101425

**Published:** 2026-03-02

**Authors:** Bruna Lopes Resende, João Sérgio da Fonseca Guimarães, Ana Luiza Amâncio Caetano, Ana Luiza Matos De Oliveira, Bruno Rezende Souza, André Gustavo Oliveira

**Affiliations:** a Laboratory of Neurodevelopment and Evolution (NeuroDEv), Department of Physiology and Biophysics, Institute of Biological Sciences, UFMG, Belo Horizonte, Brazil; b Laboratory of Physiological Circuits, Department of Physiology and Biophysics, Institute of Biological Sciences, UFMG, Belo Horizonte, Brazil; c Economic Development Unit, Economic Commission for Latin America and the Caribbean (ECLAC), Mexico City, Mexico

**Keywords:** Neurospsychiatric disorders, Mental health, Public health, Burden of diseases, Psychiatric reform

## Abstract

**Background:**

Neuropsychiatric disorders are among the leading causes of disability worldwide, generating substantial healthcare utilization and costs. Brazil, which hosts the world's largest universal public health system, implemented psychiatric reform to reduce dependence on long-term hospitalization and expand community-based services. The study aimed to assess hospitalization trends in Brazil between 2008 and 2022.

**Methods:**

We conducted a nationwide, retrospective analysis of administrative data from Brazil's Hospital Information System (SIH/SUS) covering all psychiatric admissions (ICD-10, Chapter V) from 2008 to 2022. Outcomes included admission rates, length of stay, diagnostic distribution, demographic and regional disparities, and total hospitalization costs, adjusted for inflation. Data were standardized using WHO population projections and Markov chain Monte Carlo.

**Findings:**

Psychiatric hospitalizations declined by more than 50%, from 314,686 admissions (315 per 100,000 people) in 2008 to 151,113 (151 per 100,000) in 2022. The mean length of stay fell from 45.1 to 22.1 days. Schizophrenia spectrum disorders remained the leading cause of admission but decreased substantially (from 151 to 53 per 100,000 people), while mood disorders increased particularly among female adolescents (from 12 to 16 per 100,000 people). Males were hospitalized 70% more compared to females (males: 174; females: 102 per 100,000 people in 2021), mainly for substance- (males: 37; females: 10 per 100,000 people in 2021) and alcohol-related disorders (males: 32; females: 4 per 100,000 people in 2021). Marked regional variation persisted, with slower declines in the North. Total hospitalization costs fell by over 75% between 2010 (R$1,123,798,345) and 2022 (R$290,769,856), reflecting a sustained shift from hospital-based to community-based care.

**Interpretation:**

Brazil's sustained reduction in psychiatric hospitalizations and costs demonstrates the long-term success of psychiatric reform and the expansion of community-based mental health care. However, regional and demographic inequities underscore the need for targeted investments to ensure equitable access and expand reform achievements amid policy fluctuations.

**Funding:**

CNPq, INCT, CAPES, FAPEMIG.

## Resumo

**Introdução:**

Transtornos neuropsiquiátricos figuram entre as principais causas de incapacidade global, gerando elevada demanda por serviços de saúde e custos expressivos. O Brasil, que possui o maior sistema público universal de saúde do mundo, implementou a reforma psiquiátrica para reduzir a dependência de internações de longa permanência e ampliar a rede de atenção comunitária. Este estudo teve como objetivo descrever as tendências de internações psiquiátricas no Brasil entre 2008 e 2022.

**Métodos:**

Realizamos uma análise retrospectiva, de base populacional e abrangência nacional, utilizando dados administrativos do Sistema de Informações Hospitalares do Sistema Único de Saúde (SIH/SUS), incluindo todas as internações psiquiátricas (CID-10, Capítulo V) registradas de 2008 a 2022. Os desfechos incluíram taxas de internação, tempo de permanência, distribuição diagnóstica, desigualdades demográficas e regionais e custos totais de hospitalização, ajustados pela inflação. As estimativas foram padronizadas com projeções populacionais da Organização Mundial de Saúde (OMS) e métodos de Markov chain Monte Carlo.

**Resultados:**

As internações psiquiátricas diminuíram em mais de 50%, de 314,686 internações (315 por 100,000 habitantes) em 2008 para 151,113 (151 por 100,000) em 2022. O tempo médio de permanência caiu de 45.1 para 22.1 dias. Transtornos do espectro da esquizofrenia permaneceram como a principal causa de internação, porém com queda (de 151 para 53 por 100,000 habitantes), enquanto internações por transtornos do humor aumentaram especialmente entre adolescentes do sexo feminino (de 12 para 16 por 100,000 habitantes). Em 2021, homens apresentaram taxas de internação 70% maiores que mulheres (homens: 174; mulheres: 102 por 100,000 habitantes em 2021), principalmente por transtornos relacionados ao uso de substâncias (homens: 37; mulheres: 10 por 100,000 habitantes em 2021) e de álcool (homens: 32; mulheres: 4 por 100,000 habitantes em 2021). A variação regional foi persistente, com declínio de internações mais lento no Norte. Os custos totais de hospitalização diminuíram mais de 75% entre 2010 (R$ 1,123,798,345) e 2022 (R$ 290,769,856), sugerindo uma transição sustentada do cuidado hospitalar para estratégias comunitárias.

**Interpretação:**

A queda consistente das internações psiquiátricas e dos custos no Brasil é compatível com o impacto de longo prazo da reforma psiquiátrica e com a expansão do cuidado comunitário em saúde mental. Contudo, desigualdades regionais e demográficas indicam a necessidade de investimentos direcionados para ampliar o acesso equitativo e consolidar os avanços da reforma em contextos de instabilidade de políticas públicas.

**Research in context**

**Evidence before this study**

We searched PubMed, SciELO, and Google Scholar for studies published January 2000 to July 2025, using terms related to psychiatric hospitalization, Brazil, community-based mental health, psychiatric reform, CAPS, schizophrenia, mood disorders, substance use, and health-system financing, without language restrictions. We also screened reference lists and Brazilian governmental reports. We included population-based studies reporting psychiatric hospital admissions, trends over time, or the implementation of community mental health services. Studies restricted to non-psychiatric admissions, non-SUS datasets, or non-population samples were excluded. Available evidence indicated sustained reductions in psychiatric beds and hospitalization rates following Brazil’s psychiatric reform and the expansion of community-based services. However, prior analyses typically ended in 2014 or 2019, focused on selected regions or diagnoses, lacked age standardization, and rarely examined race/ethnicity or national cost trends. Evidence quality was generally strong at the administrative level but limited by heterogeneous designs and incomplete demographic variables.

**Added value of this study**

This study provides the most comprehensive and up-to-date national evaluation of psychiatric hospitalizations in Brazil, analyzing all SUS admissions from 2008–22. It extends the literature by: (1) producing 15-year national time series spanning major policy shifts; (2) integrating age-standardized risks with detailed demographic, diagnostic, and regional disaggregation; and (3) offering a longer-term inflation-adjusted national cost analysis of psychiatric admissions. These combined approaches reveal sustained national declines in admissions and costs, alongside persistent regional, age, sex, and race/ethnicity inequities that were previously undocumented altogether at this granularity.

**Implications of all the available evidence**

Our findings, in conjunction with previous research, suggest that Brazil’s psychiatric reform has produced durable reductions in hospital dependence and expenditures, supporting the transition toward community-based care. Yet the combined evidence also shows that progress has been uneven, with slower declines in underserved regions, rising admissions for mood disorders among adolescents, and marked disparities by sex and race/ethnicity. Strengthening and equitably distributing community mental health services, ensuring stable financing, and prioritizing youth mental health are critical to sustaining reform achievements. Future work should link individual-level datasets to assess readmission patterns, treatment continuity, and the real-world effectiveness of community care models.

## Introduction

Neuropsychiatric disorders are a leading cause of disability worldwide. Their impact stems from high prevalence, early onset, functional impairment, and a tendency for recurrence.^1^ Globally, about 30% of adults will experience a mental disorder in their lifetime, with nearly 80% residing in low- and middle-income countries, where 86% of suicides also occur.^2^ In Brazil, depressive and anxiety disorders are among the top causes of years lived with disability, underscoring the need for effective mental health policies.^3^

Since the 1980s, Brazil's mental health policy has been shifting from institutional to community-based care, a change solidified by Law 10.216/2001, which prioritized services like Psychosocial Care Centers (*Centros de Atenção Psicossocial*–CAPS).^4^ This reform led to a 33.3% reduction in psychiatric beds between 2000 and 2014 in Brazil's Unified Health System (*Sistema Único de Saúde*–SUS), reflecting the policy's emphasis on reducing reliance on hospital-based care, and an expansion of CAPS coverage, which has been associated with lower hospitalization rates in some areas.^5^

Despite these advances, challenges persist. Emergency admissions constitute the majority of psychiatric hospitalizations (81.9%), and high readmission rates (the “revolving door” phenomenon) remain a significant concern.^6^ Financial constraints and uneven distribution of services exacerbate these issues, particularly in underserved regions.^7^ Since 2017, under rules of austerity, national mental health policy has undergone partial reversals. Regulatory changes formally reintegrated psychiatric hospitals into the Psychological Care Network and expanded funding for public and private hospital psychiatric admissions, including therapeutic communities (private centers for substance abuse disorder that aim for isolation and abstinence, and are often religiously affiliated), while limiting federal investment in community-based services.^7^, ^8^, ^9^, ^10^ These shifts provide important context for interpreting recent trends in the Brazilian mental health scenario.

In this study, we analyzed data from the Department of Health Informatics of SUS (DATASUS) to provide an updated and comprehensive overview of the current mental health landscape, incorporating key variables such as ethnicity, financial burden, and a broader range of age groups. Our findings are intended to inform policy interventions, highlighting areas for improvement in resource allocation and service delivery. We found a sustained decrease in psychiatric hospitalization rates and associated costs that reflect both the intended effects of community-based reform and the unintended consequences of austerity-induced policies of mental health, producing an ambivalent scenario of apparent success that may conceal unmet needs, particularly in disadvantaged regions and population groups.

## Methods

### Study design and data collection

All data used in this study were obtained through the Hospital Information System of the Unified Health System (SIH/SUS), managed by the Brazilian Ministry of Health, through the Department of Health Care, together with the State and Municipal Health Departments. All neuropsychiatric disorders analyzed are classified by the International Classification of Diseases (ICD-10) as “mental and behavioral disorders” and are grouped in chapter V (Supplementary Table S1). For this study, we analyzed: the variables, Age group, Sex, Race/ethnicity, and ICD10 list; and the parameters, Average days of stay in the hospital, Total hospitalization cost, Mean cost of hospitalization admission, and Hospitalization admission as detailed in (Supplementary Tables S2 and S3).

The hospital units participating in SUS (public or private under contract) send information on hospital authorizations to municipal or state managers. The information is, then, transferred to DATASUS by the Department of Health Informatics of SUS, forming a valuable database that comprises the whole population serviced by SUS (approximately 70% of the total population). Authorization information was retrieved from a list of neuropsychiatric disorders based on ICD codes consisting exclusively of mental and behavioral disorders (ICD information). The relative hospitalization rates of these neuropsychiatric disorders were analyzed from 2008, as it was the first available period after a significant change to the classification process adopted by SUS-participating units, to 2022 (that is, all periods available in the system after the change at the time of data retrieval). It is important to note that: we do not have access to the medical records of any patient; therefore, this administrative database does not allow tracking of disease progression, readmission of individuals, or any other individual information, thus we conducted an ecological time-series analysis of hospital admissions. This data does not include the whole burden of users of private sector care, only those paid through SUS, and thus may underestimate true rates. Cost data were retrieved from the same database as Authorizations from the morbidity section on the Tabnet platform from DATASUS, according to neuropsychiatric disorders, year, and region. The costs retrieved were solely related to the authorized hospitalization admissions and are a direct representation of the number of admissions (Supplementary Table S3). Costs were corrected by the National Extended Consumer Price Index to adjust for inflation.

The Brazilian regional divisions used in this study are the official administrative divisions of the territory; each partition was grouped according to a combination of geographical, economic, and demographic factors analyzed by the Brazilian Institute of Geography and Statistics (*Instituto Brasileiro de Geografia e Estatística*–IBGE).^11^ Brazil is divided into regions: North, Northeast, Midwest, Southeast, and South. Data on the population number were obtained from the census, or estimates carried out by IBGE throughout the analysis, and were used to obtain the SUS psychiatric hospitalization rates per 100,000 population that we referred to as Admissions by neuropsychiatric disorders (per 100,000 people) in figures and as hospitalization rates or simply rates throughout the text. For age standardization, we employed the WHO World Standard Population that projects population estimates from 2000 to 2025.^12^

### Epidemiological analysis

Age-specific hospitalization rates per 100,000 population for neuropsychiatric disorders, as well as age-standardized (age-adjusted) numbers of psychiatric admission authorizations within SUS, were calculated using age groups defined by the Brazilian Ministry of Health: 0–4, 5–9, 10–14, 15–19, 20–29, 30–39, 40–49, 50–59, 60–69, 70–79, and ≥80 years.^13^ Each age analysis was performed through the “surveil” package, using the Hamiltonian Markov chain Monte Carlo modeling algorithm to determine the age-specific hospitalization rates per 100,000 people. Posteriorly, we multiplied these data by their respective weight according to the WHO world standard population (2000–2025^12^) and aggregated to provide age-standardized number of admission authorizations per 100,000 people. This analysis was performed to allow for comparison of our data with different populations that may have different age structures than the Brazilian. Averages were calculated as a ratio of absolute values: days of stay or total hospitalization costs, divided by the total number of hospital admissions. Data were analyzed using R (version 4.4.2) and RStudio (2024.04.2 Build 764) and the data visualization was performed through “geobr” and “ggplot2” packages.

As SUS serves approximately 70% of the population and we are using the official counts provided by the government, we opted to avoid statistical testing when comparing the impact of disorders in different regions, sexes, races, and age groups.

### Ethical statement

The study relied on publicly available, unrestricted, de-identified secondary data. Therefore, under Brazilian regulations, it is exempt from registration and ethical review by the Research Ethics Committees system and the National Commission for Research Ethics, and no ethics approval number applies, in accordance with Brazilian National Health Council (CNS) Resolution No. 510/2016 (Art. 1, sole paragraph; Art. 2, item VI).

### Role of the funding source

The funding sources had no role in study design; in the collection, analysis, and interpretation of data; in the writing of the report; and in the decision to submit the paper for publication.

## Results

### Admissions and length of stay by region, disorder, sex, race/ethnicity, and age

#### Age and sex

**Fig. 1:**
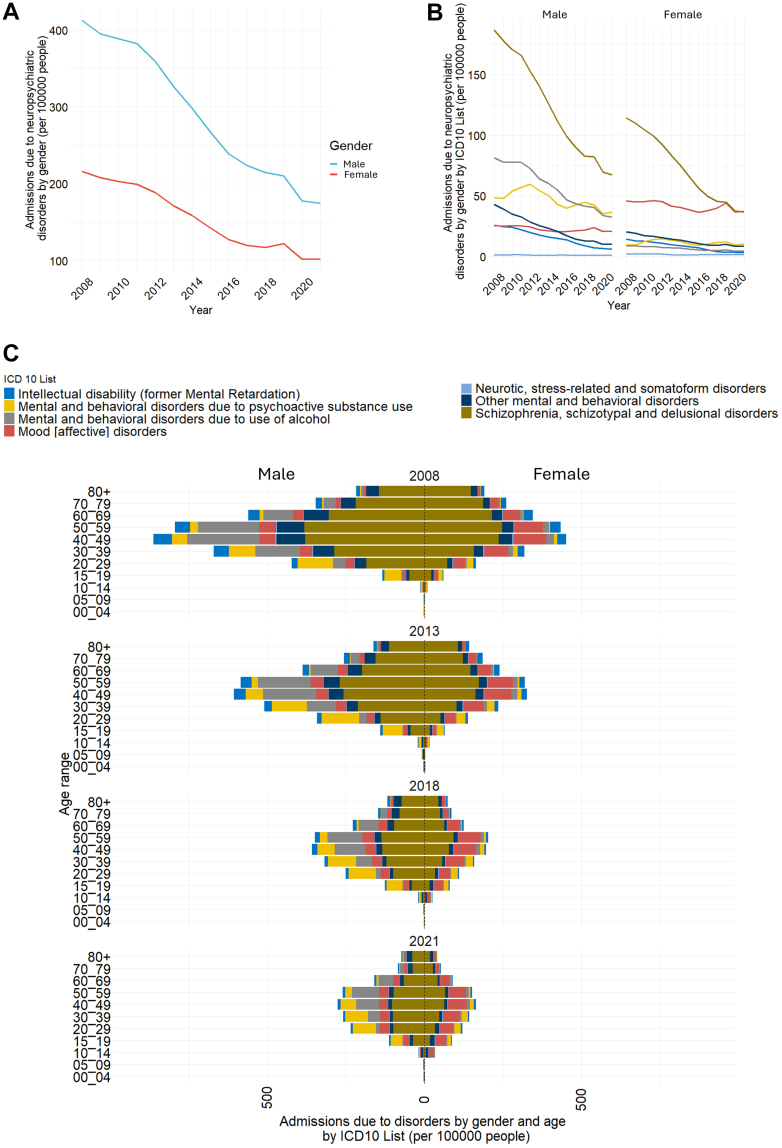
Admissions due to neuropsychiatric disorders by sex. (A) Admissions due to neuropsychiatric disorders by sex (per 100,000 people) from 2008 to 2021. Both males and females exhibited a decrease in admissions; however, males' admissions remained almost double that of females throughout the period. (B) Admissions due to neuropsychiatric disorders by sex by ICD10 classification (per 100,000 people) from 2008 to 2021. Schizophrenia, schizotypal, and delusional disorders were the main causes of admissions for both males and females. (C) Admissions due to neuropsychiatric disorders by sex, age, and ICD10 classification (per 100,000 people), 2008, 2013, 2018, 2021. The pyramid displays males' admissions to the left and females' to the right; each layer is composed of the different ICD-10 classified causes of admissions for specific age groups. From 2008 to 2021, an overall reduction of admissions has been observed in all sexes, and for most disorders and age groups.

Males were hospitalized nearly twice as often as females, though rates declined for both groups (Fig. 1A). Schizophrenia spectrum disorders were the leading cause for both sexes throughout the study (Fig. 1B and C). Among males, alcohol-related disorders were the second most frequent diagnosis until 2018, after which substance-related disorders assumed this position (Fig. 1B and C). Mood disorders rose to fourth after 2015. Admissions for most disorders declined, except mood disorders and neurotic/stress-related disorders, which remained stable (Fig. 1B and C). Among females, schizophrenia spectrum and mood disorders dominated, converging by 2018–2022 (Fig. 1B and C). Substance-related disorders rose to third after 2017, while intellectual disability fell in rank (Fig. 1B and C). Alcohol use disorders fluctuated between the fifth and sixth (Fig. 1B and C). Admissions for most diagnoses decreased, but mood disorders, substance-related disorders, and neurotic/stress-related disorders persisted at stable levels (Fig. 1B and C).

We age-standardized admissions for the four leading causes: schizophrenia spectrum disorders, mood disorders, substance-related disorders, and alcohol use disorders. Schizophrenia-related admissions fell from >150 per 100,000 in 2008 to <50 in 2022 (Supplementary Figure S1A). Hospitalization rates increased beginning at ages 15–19, peaked between 40 and 59, and remained elevated into late life. The sharpest reductions were in ages 40–79, with hospitalization rates decreasing by 70–80% (Admissions per 100,000 people decreased from 306 to 82 in ages 40–49; 311 to 81 in ages 50–59; 256 to 52 in ages 60–69; 201 to 32 in ages 70–79). In contrast, hospitalization rates among adolescents (15–19) remained stable (Supplementary Figure S4A). Substance-related disorders declined, but with fluctuations, including peaks in 2012 and 2019 (Supplementary Figure S1B). Hospitalization rates began in adolescence, peaked at 20–29, remained high through 30–39, and fell after 50. Hospitalization rates for ages 40–59 remained largely stable over time (Supplementary Figure S4B). Alcohol-related disorders also declined from 45 to 15 per 100,000 between 2008 and 2022 (Supplementary Figure S1C). Hospitalization rates rose in early adulthood, peaked at 40–49, and decreased after 60. Reductions occurred across all age groups, with the steepest drop (approximately 70%, dropping from 124 to 32 admissions per 100,000 people) in ages 40–49 (Supplementary Figure S4C). Mood disorder admissions followed a distinct trajectory. Hospitalization rates began at ages 10–14, peaked at 40–59, and declined after 70 (Supplementary Figure S1D). Substantial reductions occurred among adults aged 30–79, most notably in the ages 40–59. However, rates remained stable in ages 20–29 and rose in adolescents after 2019 (Supplementary Figure S1D; Supplementary Figure S4D).

Age and sex interactions showed clear diagnostic patterns. Schizophrenia spectrum disorders predominated across sexes from early adulthood onward, with onset of predominance shifting later over time: >15 years in 2008, >20 in 2013, >30 in 2018, and by 2021, >20 in males and >40 in females (Fig. 1C). Substance-related disorders peaked among adolescents and young adults, especially males 15–39 (Fig. 1C). Alcohol use disorders were the second leading cause in males 40–69 (Fig. 1C). Mood disorders ranked second in females 20–79, and became the leading diagnosis among females aged 10–39 by 2021 (Fig. 1C). Finally, Other behavioral disorders were most common in adults ≥80 and, by 2021, also in boys aged 10–14 (Fig. 1C).

#### Region

**Fig. 2:**
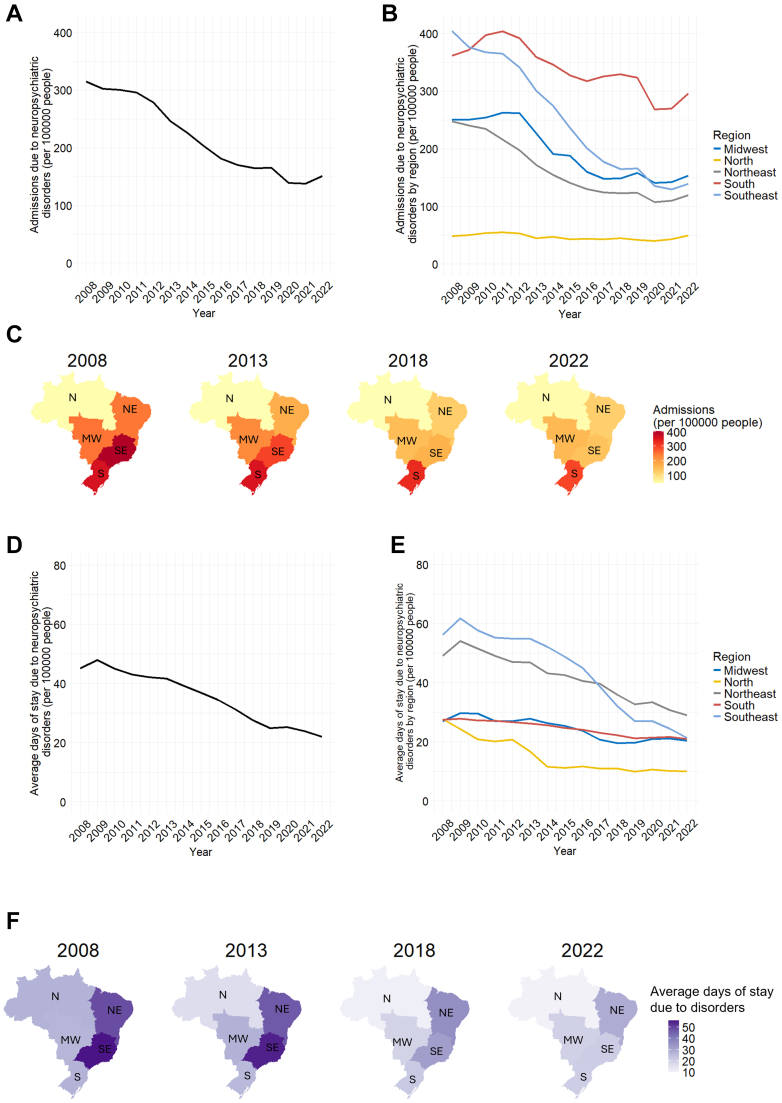
Admissions due to neuropsychiatric disorders. (A) Number of hospital admissions due to the analyzed neuropsychiatric disorders per 100,000 people from 2008 to 2022. The number of admissions was acquired per year through the DATASUS platform from the Brazilian government. It shows a steady decline from 2008 to 2022, the last year analyzed. (B) Hospital admissions per 100,000 according to Brazil's geographic regions (Midwest, North, Northeast, South, Southeast). The number of hospital admissions has been decreasing from 2008 to 2022 in the Midwest, Northeast, South, and Southeast regions. Contrastingly, the North region displayed a reduced but stable pattern during the period. (C) Comparison of the number of hospital admissions between Brazil's five regions. Maps show data of hospital admissions every five years, with admissions per 100,000 people indicated by the color bar. (D) Average days of stay due to psychiatric disorders. Data is comprised of the total days of stay due to the analyzed disorders divided by the number of hospital admissions due to these disorders. Average days of stay displayed a reduction from 2008 to 2022. (E) Average days of stay due to neuropsychiatric disorders in the five regions of Brazil. The South and Southeast regions exhibited the most pronounced reduction, the North region showed a reduction in days of stay from 2008 to 2014, and assuming a stable pattern until 2022, the Midwest and Northeast regions displayed a smaller reduction in the period. (F) Comparison of average days of stay between Brazil's five regions. Maps show data on average days of stay every five years, with average stay indicated by the color bar.

From 2008 to 2022, psychiatric hospital admissions in Brazil declined steadily, falling 52%, from 315 to 151 per 100,000 people (Fig. 2A). Age-standardized rates showed the same pattern (Supplementary Figure S2). The number of deaths due to mental disorders also showed a reduction up to 2020, followed by an increase up to 2022. The changes in death, however, occurred on a much smaller scale. They dropped 34%, ranging from 0.58 to 0.38 deaths per 100,000 people (Supplementary Figure S3A). Declines in admissions were most pronounced in the Midwest, Northeast, and Southeast regions, moderate in the South, and minimal in the North, where the already lower rates remained nearly constant throughout the period (Fig. 2B–C). The number of deaths showed a difference in regional patterns when compared to hospital admissions. Southeast region displayed the most pronounced reduction, followed by the South region. The Midwest region showed an almost constant pattern, while the North and Northeast regions had a slight increase in the number of deaths (Supplementary Figure S3B). Average length of stay also decreased, from 45.1 days in 2008 to 22.1 days in 2022 (Fig. 2D). This downward trend was observed across all regions, though reductions were less marked in the Midwest and South (Fig. 2E–F).

**Fig. 3:**
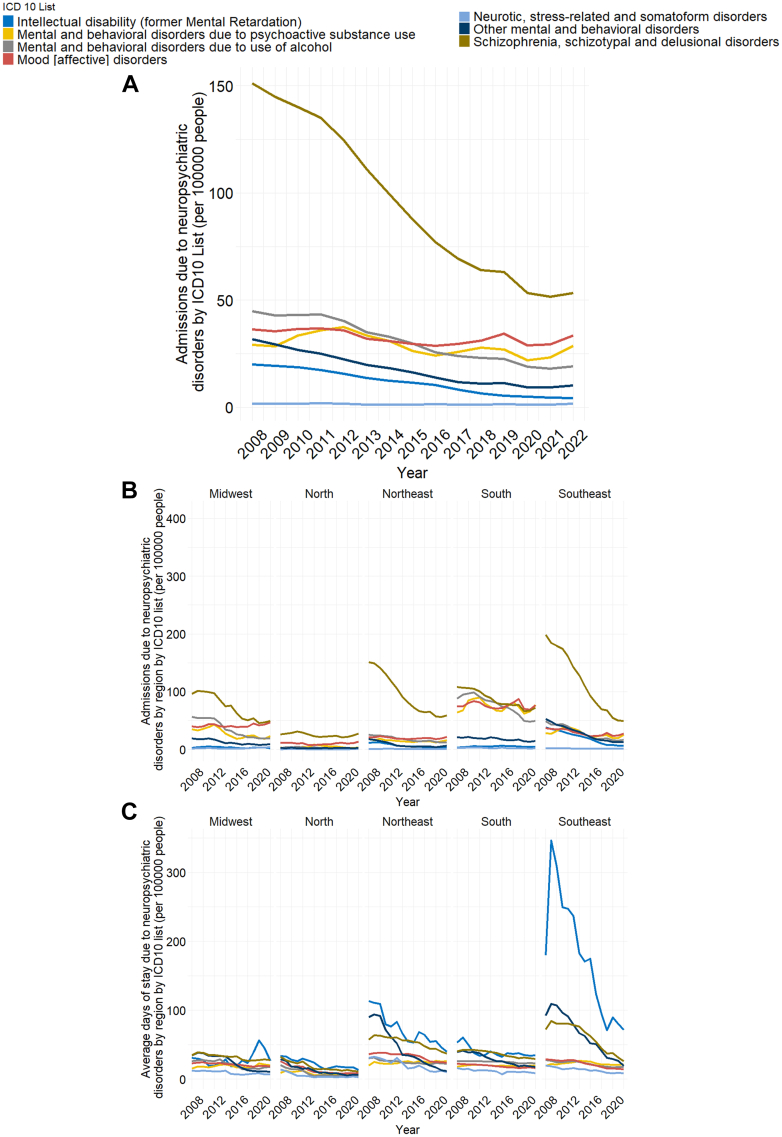
Admissions due to neuropsychiatric disorders by ICD-10 classification. (A) Number of hospital admissions due to disorders by ICD10 classification per 100,000 people. Schizophrenia, schizotypal, and delusional Disorders were the leading cause of admission throughout the period, albeit displaying a pronounced reduction from 2008 to 2022. (B) Number of hospital admissions due to disorders by region by ICD10 classification per 100,000 people. All regions displayed Schizophrenia, schizotypal, and delusional Disorders as their main cause of hospital admission, except for the South region, which had Mood [affective] disorders as the leading cause. (C) Average days of stay by region due to neuropsychiatric disorders by ICD-10 classification. The Southeast region exhibited overall higher averages of stay, especially for Intellectual disability (former Mental Retardation).

Across the study, schizophrenia spectrum disorders were the most frequent cause of admission, followed by mood disorders, substance-related disorders, alcohol use disorders, intellectual disability, neurotic/stress-related disorders, and other behavioral disorders (Fig. 3A). Regarding the number of deaths, schizophrenia also went through a reduction, while Alcohol-related disorders, the main cause of death in later years, remained almost constant (Supplementary Figure S3C). Schizophrenia remained the leading diagnosis in all regions, except in the South region, where mood disorders became the main cause between 2018 and 2022 (Fig. 3B). Deaths due to schizophrenia showed a reduction in Southeast, South, and Northeast regions, while deaths due to mood disorders showed a slight increase in these areas. The Midwest and North regions displayed less pronounced changes and noisier data (Supplementary Figure S3D). Intellectual disability accounted for the longest average stays in the North and Southeast regions, alternating with schizophrenia spectrum disorders in the Midwest, Northeast, and South (Fig. 3C).

#### Race and ethnicity

**Fig. 4:**
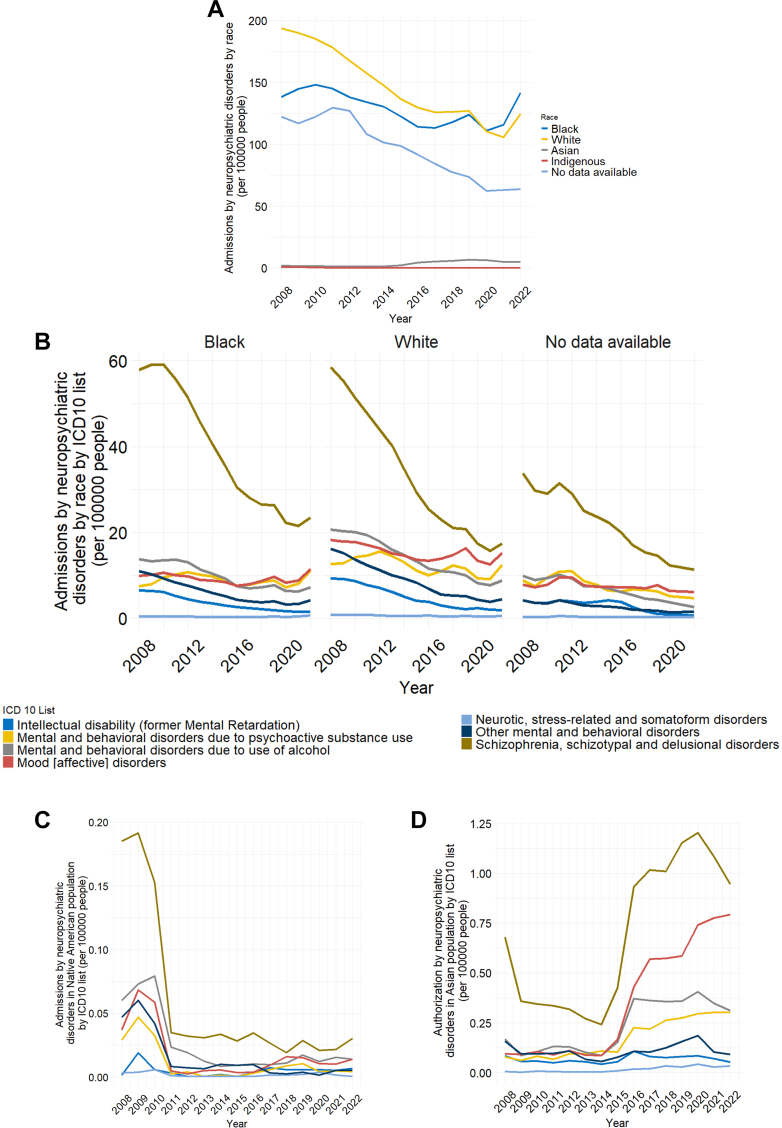
Admissions due to neuropsychiatric disorders by race/ethnicity. (A) Admissions due to neuropsychiatric disorders by race/ethnicity (per 100,000 people) from 2008 to 2022. White and Black populations were the most admitted in the period, albeit they displayed a decline in admissions throughout. The Asian population exhibited a slight increase from 2015 to 2022, and a significant portion of the population opted not to self-report their race/ethnic group. (B) Admissions due to neuropsychiatric disorders by race/ethnicity by ICD10 classification (per 100,000 people) from 2008 to 2022. Black and White populations displayed similar disorder admission profiles, with Schizophrenia, schizotypal and delusional disorders, and Mood [affective] disorders ranking as the leading cause of admissions. (C) Admissions due to neuropsychiatric disorders in the Native American population by ICD10 classification (per 100,000 people) from 2008 to 2022. The Native American population displayed a significantly lower scale of admissions since 2008, which was exacerbated by an accentuated reduction in 2011 for most disorders. (D) Admissions due to neuropsychiatric disorders in the Asian population by ICD-10 classification (per 100,000 people) from 2008 to 2022. The Asian population shows a different pattern than other groups, with an increase in admissions from 2015 to 2022 regarding the leading five causes of admission.

Admissions were highest among White and Black individuals, both showing overall declines (Admissions between 2008 and 2020 decreased from 138 to 111 admissions per 100,000 people among Black population and from 193 to 110 admissions per 100,000 people among White population) followed by a modest increase in the last two years (In 2022 rates reached 141 and 124 admissions per 100,000 people among Black and White populations respectively). Asian and Indigenous populations had the lowest absolute numbers (Fig. 4A). Schizophrenia spectrum disorders were the leading cause across all groups, with declining prevalence in most except Asians (Fig. 4B–D). Alcohol-related disorders, the second most common diagnosis in 2008, fell to fourth place by 2022 among White and Black groups, but remained second in Indigenous and third in Asian groups (Fig. 4C–D). Mood disorders rose in prominence, moving from third or fourth place in 2008 to second across all groups by 2022 (Fig. 4B–D). Substance-related disorders also increased, reaching third place in White and Black groups, though declining in rank among Indigenous and Asian populations (Fig. 4B–D). Intellectual disability, neurotic/stress-related disorders, and other behavioral disorders consistently occupied the lowest ranks (Fig. 4B–D).

### Cost associated with admissions due to neuropsychiatric disorders

**Fig. 5:**
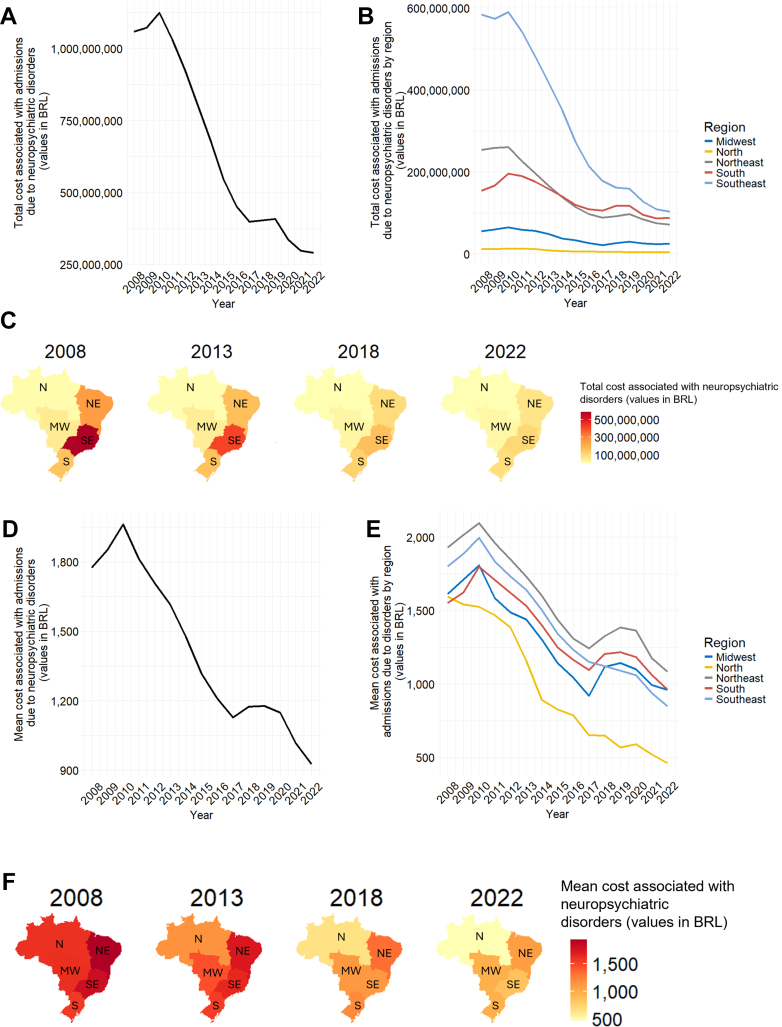
Costs associated with neuropsychiatric disorders admissions. (A) Total costs associated with neuropsychiatric disorders in Brazil from 2008 to 2022. The portion of the healthcare budget spent on hospital admissions decreased from over a billion BRL in 2010 to over a quarter of a billion BRL in 2022. (B) Total costs associated with neuropsychiatric disorders in Brazil by region from 2008 to 2022. South, Northeast, and especially Southeast regions exhibited a reduction in the expenditure associated with admission; Midwest and North regions displayed a stable, albeit low expenditure throughout the period. (C) Comparison of the total cost associated with hospital admissions between Brazil's five regions. Maps show data on hospital admissions costs every five years, with values indicated by the color bar. (D) Mean costs associated with neuropsychiatric disorders in Brazil from 2008 to 2022. Since 2010, the mean cost of hospital admission has decreased from over 1900 BRL to less than 1000 BRL. (E) Total costs associated with neuropsychiatric disorders in Brazil by region from 2008 to 2022. All regions displayed a reduction in the mean cost associated with hospital admissions. (F) Mean costs associated with neuropsychiatric disorders between Brazil's five regions. Maps show data on hospital admissions costs every five years, with values indicated by the color bar.

Between 2010 and 2022, total psychiatric hospitalization costs in Brazil fell by more than 75% (From 1,123,798,345 BRL to 290,769,856 BRL) (Fig. 5A). Declines were marked in the Northeast, South, and Southeast regions, while costs in the North and Midwest regions remained low and stable throughout (Fig. 5B and C). Mean admission costs decreased nationally from 2010 to 2017, rose slightly through 2018, and fell again by 2022 (Fig. 5D). Regional patterns mirrored national trends, except in the Southeast and North regions, where costs decreased steadily from an already lower baseline (Fig. 5E–F).

## Discussion

This study analyzed psychiatric hospitalization trends within Brazil's Unified Health System (SUS) from 2008 to 2022. We examined demographic factors (sex, ethnicity, age), financial implications, and regional variations. SUS, the world's largest universal, publicly funded healthcare system, operates under a decentralized governance model.^14^ Our findings reveal a complex dynamic: an overall decrease in hospitalization rates, yet persistent regional and demographic disparities that pose considerable challenges to the Brazilian mental health system.

Psychiatric hospital admissions and average length of stay consistently declined in Brazil from 2008 to 2022 (from 45.10 to 22.07 days). This trend aligns with previous research^7^^,^^15^ and is attributed to the expansion of CAPS and a national shift toward community-based mental healthcare.^3^^,^^15^ This evolution follows the Brazilian Psychiatric Reform, formalized by Law 10.216/2001, which promoted deinstitutionalization and the development of community-based services.^3^ The development of CAPS is a primary driver of this decline, and empirical evidence consistently associates CAPS expansion with reduced hospitalization rates, as well as improved patient satisfaction and social functioning.^15^^,^^16^

Total hospitalization costs declined by more than 75% (From 1,123,798,345 BRL to 290,769,856 BRL) over the study period, mirroring reductions in the number and length of stay. However, persistently low expenditures in the North and Midwest raise concerns about inequitable resource allocation. This budgetary shift from hospital to community services is not new. Previous work noted a nine-fold increase in community services alongside a decrease in psychiatric beds from 1995 to 2005.^17^ Accordingly, they observed a lowering of psychiatric admission costs from 95% to 49% of the budget and an increase of 15% spent on community services and medication. Nevertheless, they found a 26% decrease in overall mental health expenses, reducing the percentage of the health budget destined to mental health from 5.8% to 2.3%.^17^ Our data indicate a continued decline in the overall mental health budget allocation for psychiatric hospital admissions until 2022, without a proportional increase in funding of community services.^8^^,^^18^^,^^19^ This raises financial issues concerning the destination of funds to implement and maintain mental health services, especially considering that the coverage of community-based services is still rather precarious. It is estimated that more than 70% of the Brazilian population lives in areas with low or absent coverage of community mental health services.^20^ This ambivalent success in reform implementation, in which we see an increase in funding towards community care, though not sufficiently to meet clinical demands, indicates the need for resource allocation towards the implementation, human resources, and stabilization of community-based mental health services across the country. Psychiatric reform should not be a cost-cutting measure, and sustained investment must match policy ambitions to ensure equitable care access nationwide.^17^^,^^21^

This discussion is closely related to our findings showing persistent regional heterogeneities. Despite the general trend towards deinstitutionalization, the slower decline in hospital admissions in the South region and near stagnation in the North (which had lower rates from the beginning) likely reflects inequalities in the implementation of reform and uneven distribution of substitute care services across the country.^17^^,^^18^ Mental health resources are asymmetrically distributed, with the North and Northeast regions underserved compared to the South and Southeast regions. This disproportionate distribution of the CAPS system, overprioritizing the wealthiest regions of the country, reinforces existing geographical inequalities. Insufficient or uneven CAPS coverage, which experienced rapid growth from 2002 to 2007 but significantly slowed from 2008 to 2014, may explain the continued reliance on hospitalization in some regions.^15^ The predominance of emergency admissions indicates that hospitals remain a crucial resource during crises.^3^

Schizophrenia spectrum and delusional disorders remained the leading causes of psychiatric hospitalization, although absolute admissions decreased by nearly two-thirds. They are associated with the longest lengths of stay, underscoring the need for specialized, long-term care models. The chronic and relapsing nature of schizophrenia often leads to a “revolving door” dynamic of recurrent readmissions,^6^^,^^15^ which is exacerbated by factors like poor medication adherence, illness severity, lack of social support, and a history of multiple hospital admissions.^22^ While it is widely recognized that strong social networks are sources of emotional and practical support, people with long-term disorders (especially psychotic patients) tend to have fewer friendships and social connections, which can boost a cycle of worsening psychotic symptoms followed by social withdrawal.^23^^,^^24^ Therefore, treatment modalities that invest in the social well-being of patients with schizophrenia are effective in increasing patients’ quality of life.^25^, ^26^, ^27^ Community-based models emphasizing individualized care, continuity, and social rehabilitation may mitigate the psychological burden and the revolving-door pattern characteristic of schizophrenia.^25^

Males were hospitalized nearly twice as often as females, consistent with previous studies.^3^^,^^28^ Substance and alcohol use disorders accounted for much of this disparity, consistent with higher rates of externalizing behaviors among males.^29^ In contrast, for females, mood disorders were the second most common reason for admission. Mood disorders, albeit serious and impairing, usually progress more gradually and can be managed with outpatient care, particularly when patients engage early in treatment. Females seek outpatient care more often, facilitated by social norms that permit emotional disclosure, whereas males typically enter the system later, often at crisis points, leading to higher hospitalization rates.^30^ Thus, males’ admissions probably reflect a pattern of delayed treatment and acute hospitalization, while females carry a substantial but less visible burden of mood disorders managed outside hospitals.

White and Black individuals accounted for most psychiatric hospitalizations, both showing a general decline followed by a modest recent increase. Individuals whose ethnicity was not reported (“no data available”) constituted the third most frequent group admitted to psychiatric hospitalization. This category arises from patient privacy choices or variability in reporting practices across hospitals and can complicate efforts to fully understand ethnic disparities in mental health service utilization. However, the “no data available” group closely mirrors that of the overall population, suggesting that there is no disproportional representation of any ethnoracial group. The underrepresentation of Asian and especially Indigenous populations in hospitalization data is more likely indicative of structural and systemic barriers to healthcare access rather than lower morbidity. This is supported by the highest self-harm and suicide rates within Indigenous populations despite their lower hospitalization rates.^31^ The reduction in hospital admissions may reflect critical gaps in service delivery and reporting. Geographic isolation, cultural differences, and mistrust in institutional care may all contribute to the underutilization and limited access to psychiatric services.^32^, ^33^, ^34^ Our data point to enduring inequalities in access and utilization of mental health services among Brazil's diverse population. We evidence a need for improving data collection and availability concerning ethnic minorities, besides the demand for appropriate policies that account for these disparities and prioritize the outreach of underrepresented populations.

Diagnostic profiles varied markedly by age. Schizophrenia-related admissions rose during adolescence, peaked at ages 50–59, and remained elevated into late life, consistent with the disorder's later onset and chronic trajectory.^35^ The increase in hospitalization rates among older adults may reflect the cumulative burden of the disorder over time, with patients suffering from more severe manifestations or complications of chronic schizophrenia.^36^ Substance use disorders peaked between ages 20 and 29, while alcohol-related disorders showed a later peak at 40–49 years, highlighting the importance of preventive strategies targeting adolescents and young adults.^37^ Mood disorders presented earlier, with elevated hospitalization rates beginning at ages 10–14 and peaking in midlife. This early onset aligns with growing evidence of the increasing burden of affective disorders among adolescents. Several studies show a greater risk for internalizing mood disorders in children and adolescents than in adults.^38^ Suicide is among the leading causes of death in adolescents worldwide (WHO, Global Health Estimates 2000–2019). Extensive evidence links exposure to early-life stressors (such as neglect, abuse, grief, and low socioeconomic status) to earlier onset and greater severity of depressive and anxiety disorders, with symptoms often emerging in adolescence.^38^, ^39^, ^40^, ^41^, ^42^ During the COVID-19 pandemic, food insecurity and social isolation were associated with increased symptoms of anxiety and depression among Brazilian children and adolescents.^43^^,^^44^ Therefore, the observed increase in early mood disorder hospitalization rates may signal vulnerabilities and unmet needs for healthy development, together with social inequalities. Together, these profiles illustrate the need for age-sensitive planning in psychiatric services that, so far, have been designed to meet adult-centered needs.

Mental disorders are projected to account for an increasing proportion of disability worldwide, especially in low- and middle-income countries.^2^^,^^45^ While Brazil's reduction in psychiatric hospitalizations suggests progress in the intended effects of community-based mental health reform, we also observe unintended consequences of austerity-induced contraction in resource allocation that leads to the underfunding of CAPS and the persistent reliance on emergency admissions.^7^^,^^15^^,^^17^^,^^28^ Here, we show an ambivalent scenario in which apparent success in reform implementation may conceal unmet needs, particularly concerning disadvantaged regions and population groups.

Certain limitations must be acknowledged. Analyses relied on administrative data from SIH/SUS, which do not allow tracking of individual patient trajectories, severity, comorbidities, or treatments outside hospitalization.^3^ While linkage techniques can mitigate this for specific cohorts,^15^ our ecological-level analysis cannot establish causality at the individual level. Also, the data represents only care provided within the SUS, excluding the private sector and individuals with no care access. Brazil has a mixed public-private health system, in which approximately 70% of the population relies primarily on the public system, while 30% is covered by private health insurance (5% relying exclusively on private care), a proportion that increased gradually until 2014 and slightly declined thereafter.^46^, ^47^, ^48^ This limits generalizability to the entire population, possibly leading to underestimation of hospital admission rates and hiding possible shifts in the dynamics of public and private sectors.^3^ This can also influence regional patterns of hospitalization in public hospitals, since the access to private health insurance is lowest in the Northern region and highest in the Southern.^49^^,^^50^ However, it is worth noting that 79% of psychiatric beds in Brazil are located in state-funded institutions.^51^ Another limitation of this study is the presence of missing ethnoracial data, which may hinder the precise assessment of ethnic disparities in mental health. In addition, well-documented sex disparities in healthcare-seeking behavior, with females engaging more frequently with services, may introduce bias into population-level estimates.^52^ Finally, data quality, though generally high for mortality, may suffer from coding errors or missing information, particularly for variables like ethnicity in Indigenous populations.^53^

This study provides robust evidence of an apparent successful national transition away from prolonged psychiatric hospitalization in Brazil that reflects mental health policies, particularly the Psychiatric Reform and the expansion of community services. However, this transition is far from complete. We highlight persistent disparities in regional, ethnic, and age-related inequality of service implementation and access, aggravated by austerity-induced policies decreasing funding for expansion of community-based services and potentially reasserting hospital-centric models of psychiatric care. This ambivalent success of reform implementation may conceal regressive policies and unmet needs, particularly in regions and ethnic groups that are historically underserved. These findings emphasize the need for targeted, equitable investment in community-based mental health services and highlight priority areas for policy development. Sustained efforts will be essential to address the growing burden of neuropsychiatric disorders.

## Contributors

AGO conceived the idea of the analysis, with input from all authors. JSFG and ALAC collected and analyzed all the data in this study, with input from ALMO, BRS, and AGO. BLR, JSFG, BRS, and AGO interpreted the results with contributions from all authors. ALMO conducted the corrections of costs based on the national extended consumer price index throughout the years. ALAC and AGO have directly accessed and verified the data. BLR, JSFG, BRS, and AGO produced the submitted version of the manuscript, with significant contributions from all authors.

## Data sharing statement

The findings of this study are supported by data available at public online repositories maintained by the Brazilian Federal government. The system allows anyone who wishes to access the data for any purpose (https://datasus.saude.gov.br/informacoes-de-saude-tabnet/). Data can be retrieved as follows: Morbidity (http://tabnet.datasus.gov.br/cgi/deftohtm.exe?sih/cnv/niuf.def), Mortality (http://tabnet.datasus.gov.br/cgi/deftohtm.exe?sim/cnv/obt10uf.def).

## Editor note

The Lancet Group takes a neutral position with respect to territorial claims in published maps and institutional affiliations.

## Declaration of interests

Authors declare no competing interests.

## References

[1] Ferrari A.J., Santomauro D.F., Aali A. (2024). Global incidence, prevalence, years lived with disability (YLDs), disability-adjusted life-years (DALYs), and healthy life expectancy (HALE) for 371 diseases and injuries in 204 countries and territories and 811 subnational locations, 1990–2021: a systema. Lancet.

[2] Steel Z., Marnane C., Iranpour C. (2014). The global prevalence of common mental disorders: a systematic review and meta-analysis 1980–2013. Int J Epidemiol.

[3] Silva M.G.D., Daros G.C., Bitencourt R.M.D., Iser B.P.M. (2021). Psychiatric hospitalizations in Brazil: exploratory and trend analysis from 2009 to 2019. J Bras Psiquiatr.

[4] Ramos A., Castaldelli-Maia J.M. (2025). Key features and current challenges of the Brazilian Psychosocial Care Centers for alcohol and other drugs (CAPS-AD). Int Rev Psychiatry.

[5] Miliauskas C.R., Faus D.P., Junkes L., Rodrigues R.B., Junger W. (2019). Associação entre internações psiquiátricas, cobertura de CAPS e atenção básica em regiões metropolitanas do RJ e SP, Brasil. Cien Saude Colet.

[6] Zanardo G.L.D.P., Moro L.M., Ferreira G.S., Rocha K.B. (2018). Factors associated with psychiatric readmissions: a systematic review. Paid (Ribeirão Preto).

[7] Sade R.M.S., Sashidharan S.P., Silva M.D.N.R.M.D.O. (2021). Paths and detours in the trajectory of the Brazilian psychiatric reform. Salud Colect.

[8] Almeida J.M.C.D. (2019). Política de saúde mental no Brasil: o que está em jogo nas mudanças em curso. Cad Saude Publica.

[9] Nunes M.D.O., Lima Júnior J.M.D., Portugal C.M., Torrenté M.D. (2019). Reforma e contrarreforma psiquiátrica: análise de uma crise sociopolítica e sanitária a nível nacional e regional. Cien Saude Colet.

[10] Lima F.A.C.D., Cabral M.P.G., Gussi A.F., Araújo C.E.L. (2023). Digressões da Reforma Psiquiátrica brasileira na conformação da Nova Política de Saúde Mental. Physis.

[11] (1970). DECRETO N^o^ 67.647, DE 23 DE NOVEMBRO DE 1970. Diário Oficial da União. https://www2.camara.leg.br/legin/fed/decret/1970-1979/decreto-67647-23-novembro-1970-409148-publicacaooriginal-1-pe.html.

[12] Ahmad O.B., Boschi-Pinto C., Lopez A.D., Murray C.J., Lozano R., Inoue M. (2001).

[13] Saúde M.D. DATASUS: População residente - notas técnicas. http://tabnet.datasus.gov.br/cgi/Ibge/popdescr.htm.

[14] Ortega F., Pele A. (2023). Brazil's unified health system: 35 years and future challenges. Lancet Reg Health Am.

[15] Rocha H.A.D., Reis I.A., Santos M.A.D.C., Melo A.P.S., Cherchiglia M.L. (2021). Internações psiquiátricas pelo Sistema Único de Saúde no Brasil ocorridas entre 2000 e 2014. Rev Saude Publica.

[16] Dalton-Locke C., Marston L., McPherson P., Killaspy H. (2020). The effectiveness of mental health rehabilitation services: a systematic review and narrative synthesis. Front Psychiatry.

[17] Andreoli S.B., Almeida-Filho N., Martin D., Mateus M.D.M.L., Mari J.D.J. (2007). Is psychiatric reform a strategy for reducing the mental health budget? The case of Brazil. Braz J Psychiatry.

[18] Trapé T.L., Campos R.O. (2017). The mental health care model in Brazil: analyses of the funding, governance processes, and mechanisms of assessment. Rev Saude Publica.

[19] Maia M.P.D.M., Severo A.K.D.S., De Medeiros W.R. (2022). Oferta de serviços e recursos humanos da Rede de Atenção Psicossocial no Brasil. Rev Psicol e Saúde.

[20] Fernandes C.J., Lima A.F.D., Oliveira P.R.S.D., Santos W.S.D. (2020). Índice de Cobertura Assistencial da Rede de Atenção Psicossocial (iRAPS) como ferramenta de análise crítica da reforma psiquiátrica brasileira. Cad Saude Publica.

[21] Patel V., Saxena S., Lund C. (2023). Transforming mental health systems globally: principles and policy recommendations. Lancet.

[22] Ferrari A.J., Charlson F.J., Norman R.E., Hay P.J. (2013). Burden of depressive disorders by country, sex, age, and year: findings from the global burden of disease study 2010. PLoS Med.

[23] Gayer-Anderson C., Morgan C. (2013). Social networks, support and early psychosis: a systematic review. Epidemiol Psychiatr Sci.

[24] Morin F.N., Mitchell E., Dhir A., Jones A. (2017). Social support: a useful tool in the management of psychotic disorders. UBC Med J.

[25] Černe Kolarič J., Plemenitaš Ilješ A., Kraner D. (2024). Long-term impact of community psychiatric care on quality of life amongst people living with schizophrenia: a systematic review. Healthcare.

[26] Giacco D., McCabe R., Kallert T., Hansson L., Fiorillo A., Priebe S., Zhang X.Y. (2012). Friends and symptom dimensions in patients with psychosis: a pooled analysis. PLoS One.

[27] Gowda G.S., Isaac M.K. (2022). Models of care of schizophrenia in the community—an international perspective. Curr Psychiatry Rep.

[28] Guimarães C.M., Cherchiglia M.L., da Rocha H.A., Braga S.F.M., Melo A.P.S. (2022). Factors associated with risk of death by suicide after psychiatric hospitalization by the unified Health System in Brazil (2002–2015). Gen Hosp Psychiatry.

[29] Eaton N.R., Keyes K.M., Krueger R.F. (2012). An invariant dimensional liability model of gender differences in mental disorder prevalence: evidence from a national sample. J Abnorm Psychol.

[30] Gagné S., Vasiliadis H.M., Préville M. (2014). Gender differences in general and specialty outpatient mental health service use for depression. BMC Psychiatry.

[31] Oliveira Alves F.J., Fialho E., Paiva de Araújo J.A. (2024). The rising trends of self-harm in Brazil: an ecological analysis of notifications, hospitalisations, and mortality between 2011 and 2022. Lancet Reg Health Am.

[32] Abarca-Brown G., Ortega F. (2024). A historical perspective on structural-based mental health approaches in Latin America: the Chilean and Brazilian cases. Crit Public Health.

[33] Borges M.F.D.S.O., Silva I.F.D., Koifman R. (2020). Histórico social, demográfico e de saúde dos povos indígenas do estado do Acre, Brasil. Cien Saude Colet.

[34] Ortega F., Wenceslau L.D. (2020). Challenges for implementing a global mental health agenda in Brazil: the “silencing” of culture. Transcult Psychiatry.

[35] Harvey P.D., Loewenstein D.A., Czaja S.J. (2013). Hospitalization and psychosis: influences on the course of cognition and everyday functioning in people with schizophrenia. Neurobiol Dis.

[36] Fornazari C., Canfield M., Laranjeira R. (2022). Real world evidence in involuntary psychiatric hospitalizations: 64,685 cases. Braz J Psychiatry.

[37] Chadi N., Bagley S.M., Hadland S.E. (2018). Addressing adolescents' and young adults' substance use disorders. Med Clin North Am.

[38] LeMoult J., Humphreys K.L., Tracy A., Hoffmeister J.A., Ip E., Gotlib I.H. (2020). Meta-analysis: exposure to early life stress and risk for depression in childhood and adolescence. J Am Acad Child Adolesc Psychiatry.

[39] Felitti V.J., Anda R.F., Nordenberg D. (2019). Reprint of: relationship of childhood abuse and household dysfunction to many of the leading causes of death in adults: the Adverse Childhood Experiences (ACE) Study. Am J Prev Med.

[40] Björkenstam E., Vinnerljung B., Hjern A. (2017). Impact of childhood adversities on depression in early adulthood: a longitudinal cohort study of 478,141 individuals in Sweden. J Affect Disord.

[41] Li M., D'Arcy C., Meng X. (2016). Maltreatment in childhood substantially increases the risk of adult depression and anxiety in prospective cohort studies: systematic review, meta-analysis, and proportional attributable fractions. Psychol Med.

[42] Infurna M.R., Reichl C., Parzer P., Schimmenti A., Bifulco A., Kaess M. (2016). Associations between depression and specific childhood experiences of abuse and neglect: a meta-analysis. J Affect Disord.

[43] Martins-Filho P.R., Quintans-Júnior L.J., de Souza Araújo A.A. (2021). Socio-economic inequalities and COVID-19 incidence and mortality in Brazilian children: a nationwide register-based study. Public Health.

[44] Zuccolo P.F., Casella C.B., Fatori D. (2023). Children and adolescents' emotional problems during the COVID-19 pandemic in Brazil. Eur Child Adolesc Psychiatry.

[45] Vollset S.E., Ababneh H.S., Abate Y.H. (2024). Burden of disease scenarios for 204 countries and territories, 2022–2050: a forecasting analysis for the Global Burden of Disease Study 2021. Lancet.

[46] Cruz L., Lima A.F.D.S., Graeff-Martins A. (2013). REVIEW ARTICLE mental health economics: insights from Brazil. J Ment Health.

[47] Massuda A., Hone T., Leles F.A.G., de Castro M.C., Atun R. (2018). The Brazilian health system at crossroads: progress, crisis and resilience. BMJ Glob Health.

[48] Dalgalarrondo P., Oda A.M.G.R., Onocko-Campos R.T., Banzato C.E.M. (2023). Challenges facing the psychiatric reform and mental health care in Brazil: critical unmet needs and prospects for better integrating the public and university sectors. SSM Ment Health.

[49] Castro M.C., Massuda A., Almeida G. (2019). Brazil's unified health system: the first 30 years and prospects for the future. Lancet.

[50] Coube M., Nikoloski Z., Mrejen M., Mossialos E. (2023). Persistent inequalities in health care services utilisation in Brazil (1998–2019). Int J Equity Health.

[51] Botega N.J. (2002). Psychiatric units in Brazilian general hospitals: a growing philanthropic field. Int J Soc Psychiatry.

[52] Gabriela Torino D.R., Giovanna Akemi Y., Luiza Malosti M., Cristiana Maria de Araújo Soares G., Natália Abou Hala N. (2023). Profile of users of the largest public health system in the world. Int Arch Nurs Heal Care.

[53] Lima E.E.C.D., Queiroz B.L. (2014). Evolution of the deaths registry system in Brazil: associations with changes in the mortality profile, under-registration of death counts, and ill-defined causes of death. Cad Saude Publica.

