# Supplementary material for: Nationwide decline in psychiatric hospitalizations and costs in Brazil, 2008–2022: a retrospective descriptive study evidencing reform-driven community mental health gains
Source: Lancet Reg Health Am. 2026 Mar 2;56:101425. doi: 10.1016/j.lana.2026.101425 (PMC12968424; doi:10.1016/j.lana.2026.101425)
Supplement: Supplementary Figures and Tables [file mmc1.docx]

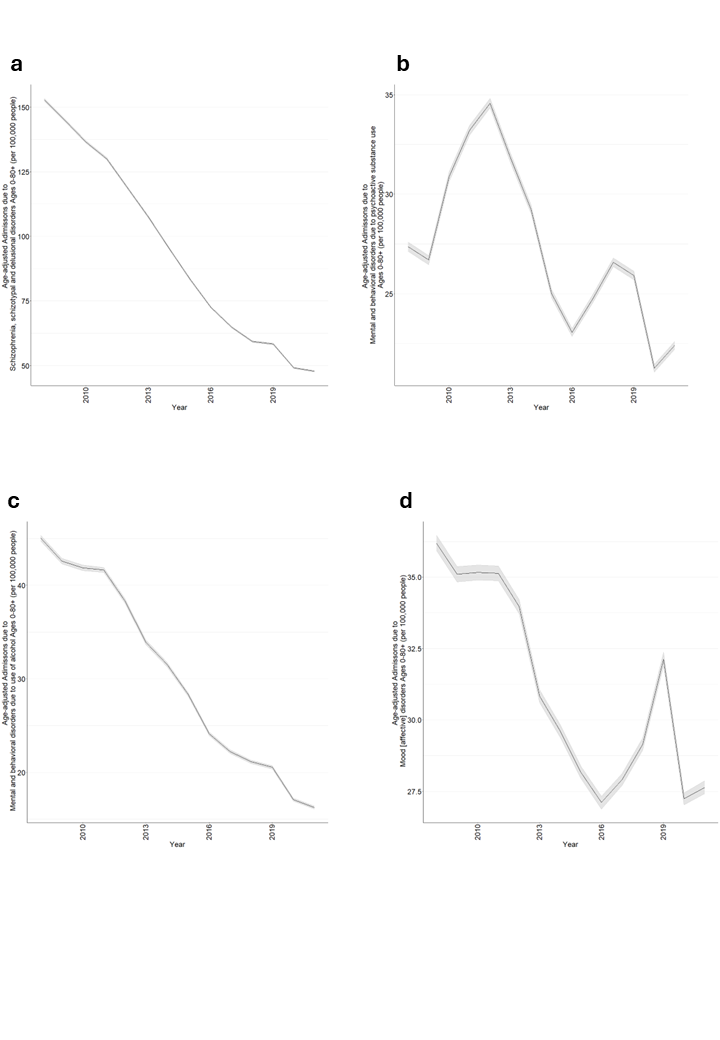


Supplementary Figure 1: Age-adjusted admissions due to the four leading admissions-cause neuropsychiatric disorders. (a) Age-adjusted admissions due to Schizophrenia, schizotypal, and delusional disorders in Brazil (per 100,000 people) from 2008 to 2021. Schizophrenia, schizotypal, and delusional disorders showed a pronounced decrease in hospital admissions from 2008 to 2021, going from approximately 150 admissions to 50 in 2021. (b) Age-adjusted admissions due to Mental and behavioral disorders due to psychoactive substances (per 100,000 people) in Brazil from 2008 to 2021. Although admissions exhibited an overall reduction from 2008 to 2021, two peaks of admissions could be observed, one in 2012 and another in 2019. (c) Age-adjusted admissions due to Mental and behavioral disorders due to the use of alcohol (per 100,000 people) in Brazil from 2008 to 2021. Admissions reduced from 45 admissions in 2008 to less than 20 in 2021. (d) Age-adjusted admissions due to Mood [affective] disorders (per 100,000 people) in Brazil from 2008 to 2021. Mood [affective] disorders showed a reduction of approximately 25% in their admissions from 2008 to 2021; however, admissions spiked in 2019. All results are shown as the means of the respective marginal probability distributions as lines and the limits of their central 95% quantile intervals as the shaded area.


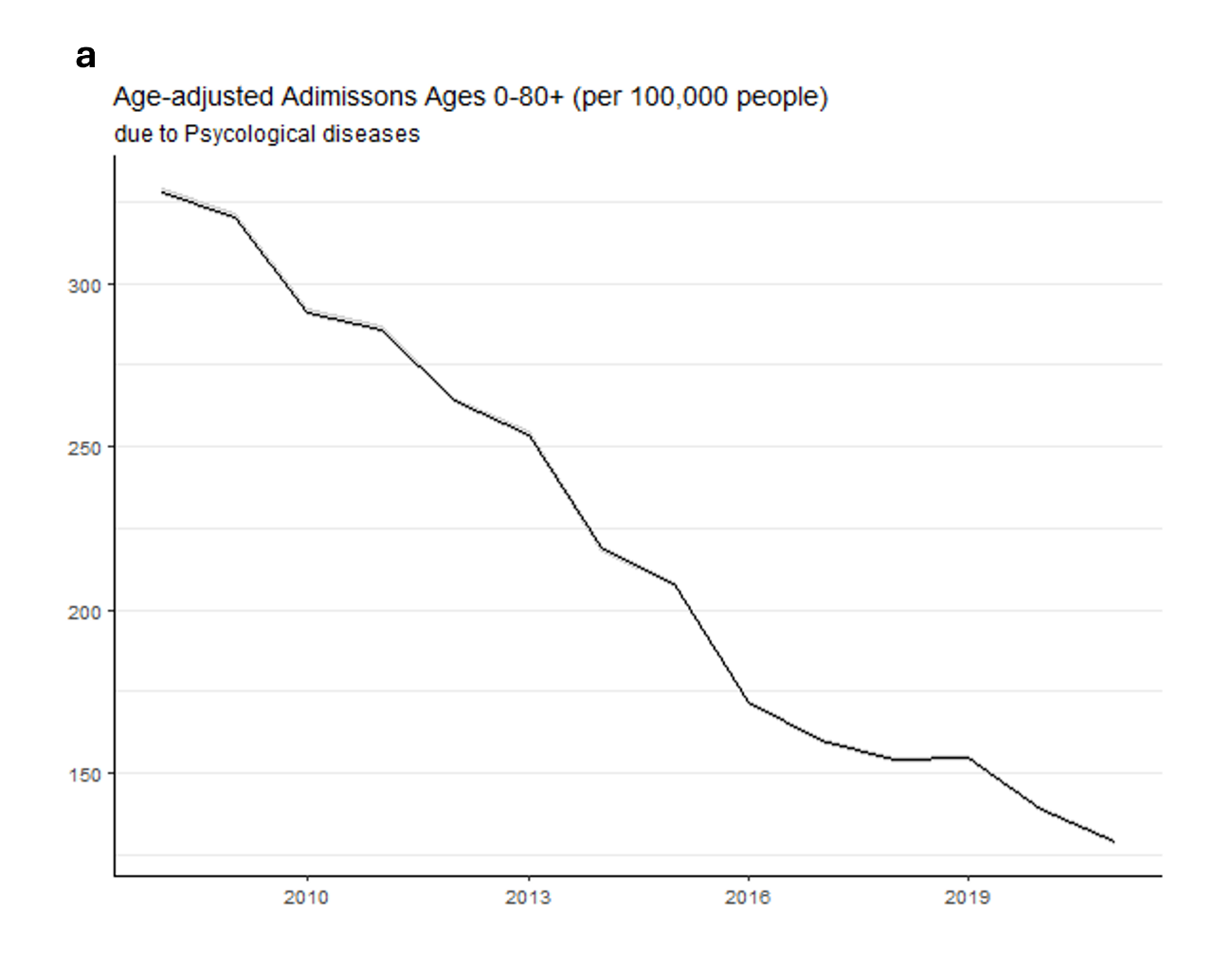


Supplementary Figure 2: Age-adjusted admissions due to neuropsychiatric disorders. (a) Age-adjusted number of admissions due to neuropsychiatric disorders in Brazil (per 100,000 people) from 2008 to 2021. The age-adjusted admission dynamic shows a continuous decrease in admissions from over 300 admissions in 2008 to less than 150 in 2021. Results are shown as the means of the respective marginal probability distributions as lines and the limits of their central 95% quantile intervals as the shaded area.


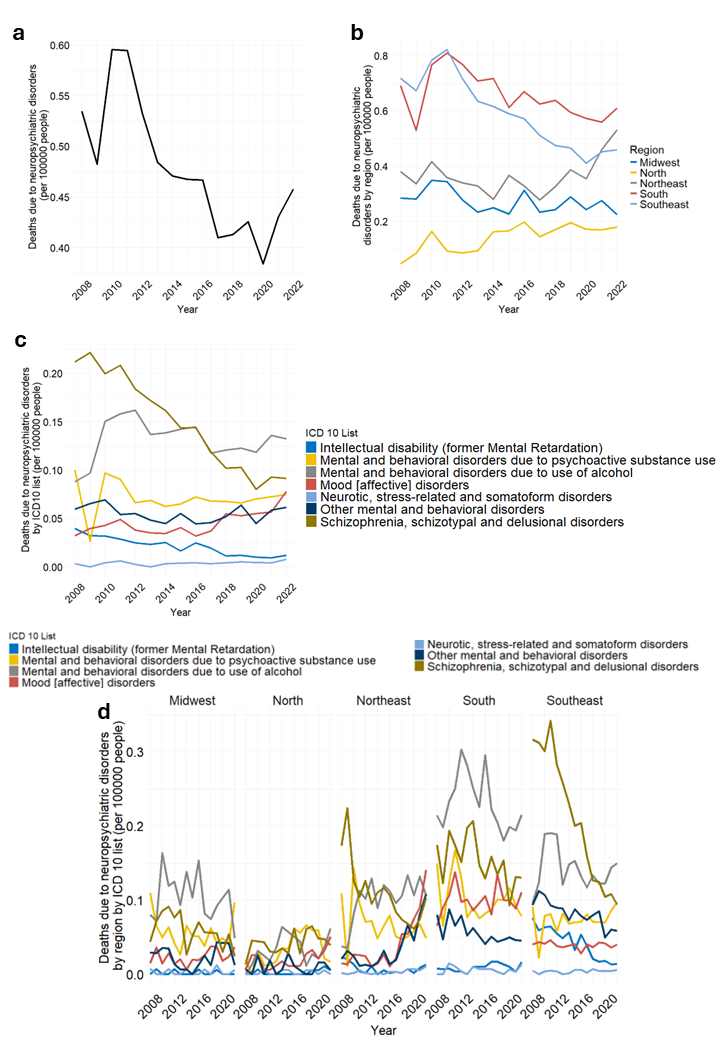

Supplementary Figure 3: Deaths due to neuropsychiatric disorders in Brazil. (a) Number of deaths per 100,000 people in Brazil due to neuropsychiatric disorders from 2008 to 2022. The number of deaths reduced from 0.58 deaths per 100,000 in 2010 to 0.38 in 2022. (b) Number of deaths per 100,000 people in Brazilian regions due to neuropsychiatric disorders from 2008 to 2022. The Southeast and South regions displayed a reduction in their number of deaths from 2008 to 2022; while the Midwest regions showed a more stable dynamic; and the North and Northeast regions showed a slight increase in the number of deaths per 100,000 people. (c) Number of deaths per 100,000 people in Brazil due to neuropsychiatric disorders by ICD-10 list from 2008 to 2022. Schizophrenia, schizotypal, and delusional disorders were the leading cause of death for half of the past decade, when Mental and behavioral disorders due to the use of alcohol became the leading cause of death among neuropsychiatric disorders. (d) Number of deaths per 100,000 people due to neuropsychiatric disorders by region by ICD-10 list from 2008 to 2022. The Southeast and South regions had Schizophrenia, schizotypal, and delusional disorders, and disorders due to the use of alcohol as their leading causes of death through the period. The other regions had less clear main causes of death as they oscillated more during the period, partially because of noisy data.


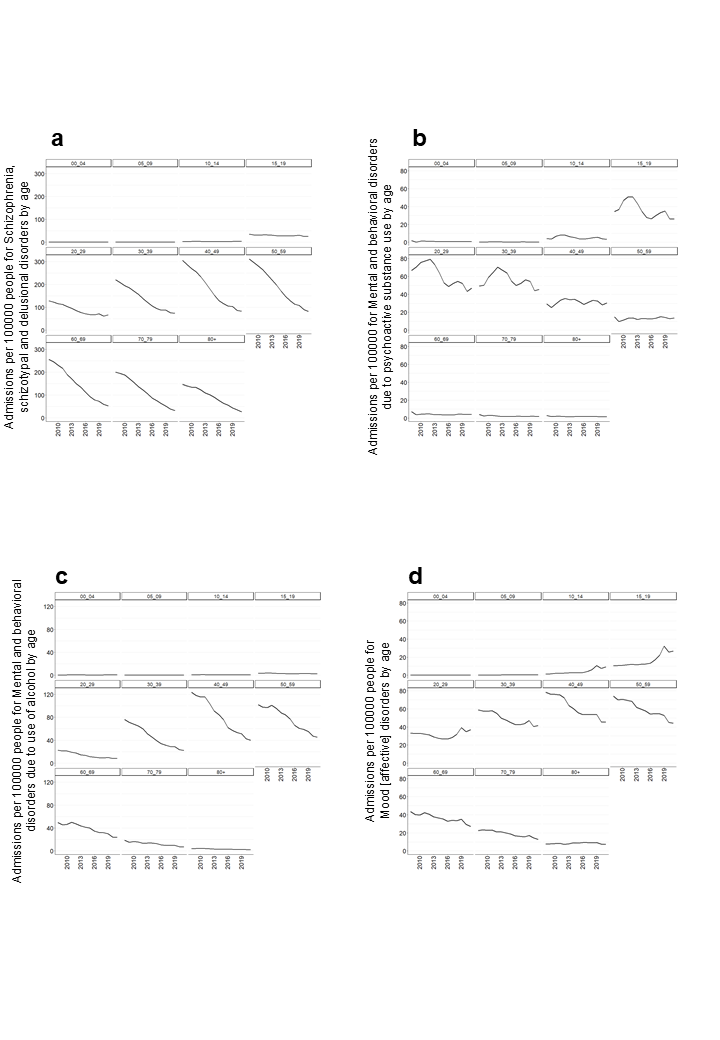


Supplementary Figure 4: Risk of admission by age among the top four neuropsychiatric causes of admissions. (a) Risk of admission for Schizophrenia, schizotypal, and delusional disorders by age from 2008 to 2021. The risk of hospital admission starts to rise at 20-29 years and remains high up to 80+ years old. (b) Risk of admission for Mental and behavioral disorders due to psychoactive substance use by age from 2008 to 2021. The risk of admission is more pronounced from ages 15-49 years old and displays two distinct peaks, one in 2012 and a smaller one in 2019. (c) Risk of admission for Mental and behavioral disorders due to the use of alcohol by age from 2008 to 2021. The risk of admission starts to rise at 20-29 years and starts to fall at 60-69 years. (d) Risk of admission for Mood [affective] disorders by age. Risk of admission starts to rise at 10-14 years, with a main increase in 2016.

**Tables**

**Table 1: Retrieved Disorders and Classification of Diseases (ICD-10)**

| **Disorder as retrieved from DATASUS** | **Included ICD-10 codes** |
| --- | --- |
| Intellectual disability (former Mental Retardation) | F70-F79 |
| Mental and behavioral disorders due to psychoactive substance use | F11-F19 |
| Mental and behavioral disorders due to the use of alcohol | F10 |
| Mood [affective] disorders | F30-F39 |
| Neurotic, stress-related, and somatoform disorders | F40-F48 |
| Other mental and behavioral disorders | F04-F09, F50-F69, F80-F99 |
| Schizophrenia, schizotypal, and delusional disorders | F20-F29 |

**Table 2: Analyzed Variables**

| **Variable** | **Description** |
| --- | --- |
| Age group | Under 1 year, 1–4 years, 5–9 years, 10–14 years, 15–19 years, 20–29 years, 30–39 years, 40–49 years, 50–59 years, 60–69 years, 70–79 years, 80 years and older. |
| Sex | Sex of the patient (male, female) |
| Race/ethnicity | Race/ethnicity of the patient (White, Black, Brown, Asian, Indigenous, or No data available) |
| ICD-10 list | The cause of hospitalization, according to the International Classification of Diseases (ICD), retrieved by list. |

**Table 3: Description of Parameters Analyzed**

| **Parameters** | **Description** |
| --- | --- |
| Average days of  stay in the hospital | Average length of stay for hospitalizations related to approved HAA (Hospital Admission Authorization), recorded as admissions, during the period. |
| Total hospitalization cost | The value refers to the approved HAA (Hospital Admission Authorization) during the period. This value may not necessarily correspond to the amount transferred to the healthcare facility, as it depends on the situation of the units. They may receive budgetary resources, or there may be retentions and payments of incentives, which are not presented here. Therefore, this value should be considered as the approved production value. |
| Mean cost of hospitalization admission | Total hospitalization cost divided by the number of hospitalizations |
| Hospitalization admission | Number of HAA (Hospital Admission Authorization) approved during the period, excluding extensions (long-stay cases). This is an approximate value of hospitalizations, as transfers and readmissions are included in this count. |
